# Evaluating cardiovascular disease risk stratification using multiple-polygenic risk scores and pooled cohort equations: insights from a 17-year longitudinal Korean cohort study

**DOI:** 10.3389/fgene.2024.1364993

**Published:** 2024-03-28

**Authors:** Yi Seul Park, Hye-Mi Jang, Ji Hye Park, Bong-Jo Kim, Hyun-Young Park, Young Jin Kim

**Affiliations:** ^1^ Division of Genome Science, Department of Precision Medicine, National Institute of Health, Cheongju-si, Republic of Korea; ^2^ National Institute of Health, Cheongju-si, Republic of Korea

**Keywords:** cardiovascular disease, multi-polygenic risk scores, pooled cohort equations, multi-PRS, PCE

## Abstract

Cardiovascular disease (CVD) remains the leading cause of mortality worldwide, caused by a complex interplay of genetic and environmental factors. This study aimed to evaluate the combined efficacy of multi-polygenic risk scores and pooled cohort equations (PCE) for predicting future CVD risks in the Korean population. In this longitudinal study, 7,612 individuals from the Ansan and Ansung cohorts were analyzed over a 17-year follow-up period. The participants were genotyped using the Korea Biobank Array, and quality-controlled genetic data were subjected to imputation analysis. The weighted sum of the PRSs (wPRSsum) was calculated using PRS-CS with summary statistics from myocardial infarction, ischemic stroke, coronary artery disease, and hypertension genome-wide association studies. The recalibrated PCE was used to assess clinical risk, and the participants were stratified into risk groups based on the wPRSsum and PCE. Associations between these risk scores and incident CVD were evaluated using Cox proportional hazards models and Kaplan–Meier analysis. The wPRSsum approach showed a significant association with incident CVD (HR = 1.15, *p* = 7.49 × 10^−5^), and the top 20% high-risk genetic group had an HR of 1.50 (*p* = 5.04 × 10^−4^). The recalibrated PCE effectively differentiated between the low and high 10-year CVD risk groups, with a marked difference in survival rates. The predictive models constructed using the wPRSsum and PCE demonstrated a slight improvement in prediction accuracy, particularly among males aged <55 years (C-index = 0.640). We demonstrated that while the integration of wPRSsum with PCE did not significantly outperform the PCE-only model (C-index: 0.703 for combined and 0.704 for PCE-only), it provided enhanced stratification of CVD risk. The highest risk group, identified through the combination of high wPRSsum and PCE scores, exhibited an HR of 4.99 for incident CVD (*p* = 1.45 × 10^−15^). These findings highlight the potential of integrating genetic risk assessments with traditional clinical tools for effective CVD risk stratification. Although the addition of wPRSsum to the PCE provided a marginal predictive improvement, it proved valuable in identifying high-risk individuals and supporting personalized treatment strategies. This study reinforces the utility of multi-PRS in conjunction with clinical risk assessment tools, paving the way for more tailored approaches for CVD prevention and management in diverse populations.

## 1 Introduction

Cardiovascular disease (CVD) is the primary cause of mortality and a significant contributor to global disease burden (Vaduganathan et al., 2022). The key to effectively managing CVD is early identification and prevention of the disease in high-risk individuals (Zhou et al., 2019; Lee et al., 2021; Vaduganathan et al., 2022). The complexity of CVD, influenced by the interplay between genetic and environmental factors, has led to numerous efforts to develop robust predictive models. These models integrate clinical variables, genetic predispositions, and lifestyle factors, such as diet and physical activity, to identify individuals at elevated risk (Wilson et al., 1998; Goff et al., 2014; Jung et al., 2015; Hippisley-Cox et al., 2017; Bae et al., 2020; Elliott et al., 2020; Lu et al., 2021; Patel et al., 2023; Yun et al., 2023).

Various risk assessment tools are integral to the diagnosis and prevention of CVD. These tools consider clinical variables and risk factors, including age, sex, blood lipid levels, blood pressure, smoking status, and diabetes mellitus (Wilson et al., 1998; Goff et al., 2014; Jung et al., 2015; Hippisley-Cox et al., 2017; Bae et al., 2020). For instance, the Framingham risk score was designed to predict the 10-year risk of ischemic heart disease (IHD) in the United States (Wilson et al., 1998). Similarly, the pooled cohort equations (PCE) provide risk assessments for IHD and stroke in the U.S., including 10-year lifetime and optimal risk evaluations (Goff et al., 2014). The United Kingdom employs QRISK for 10-year risk prediction of IHD and stroke (Hippisley-Cox et al., 2017). Moreover, Korea has developed its models for similar assessments (Jung et al., 2015; Bae et al., 2020). Based on the risks estimated by these models, healthcare providers can offer appropriate therapeutic interventions to individuals at significant 10-year disease risk.

The hereditary aspect of CVD, which is estimated to contribute to approximately 40%–60% of the risk of the disease (Zdravkovic et al., 2002; Zdravkovic et al., 2007; Tada et al., 2022), has been the focal point of recent genetic research. Over the past decade, genome-wide association studies (GWASs) have identified numerous loci associated with CVD and its risk factors (Tada et al., 2022). This led to the development of the polygenic risk score (PRS), an approach that aggregates the risks of various genetic variants, each weighted by its effect size on the disease in question (Khera et al., 2018). High-ranking individuals in the PRS distributions have been observed to have a markedly increased disease prevalence (Khera et al., 2018). The evolution of PRS into strategies that incorporate multiple PRSs (multi-PRS) simultaneously, considering the heterogeneous nature of diseases, marks a significant advancement, offering improved predictive accuracy (Lu et al., 2021; Patel et al., 2023).

Despite these advancements, the clinical application of the PRS is still in the early stages (Hao et al., 2022). The Genomic Medicine at Veterans Affairs Study has incorporated the PRS into clinical practice and developed comprehensive guidelines for its use (Hao et al., 2022). However, the majority of GWASs and subsequent PRS developments have been based on populations of European descent, potentially limiting the accuracy of these scores in non-European populations (Martin et al., 2019). Established clinical risk assessment tools also face challenges in terms of universal applicability, often requiring recalibration to suit different environmental and lifestyle factors prevalent in various populations (Jung et al., 2015; Bae et al., 2020). This underscores the need for continuous and comprehensive studies regarding PRS and clinical risk assessment tools that consider the diversity and specific characteristics of different populations, along with the rapidly evolving landscape of risk assessment methods, such as the multi-PRS approach (Elliott et al., 2020; Lu et al., 2021; Ramírez et al., 2022; Patel et al., 2023; Yun et al., 2023).

Our study aimed to evaluate the utility of combining multi-PRS with established risk assessment tools, such as the PCE, for predicting future CVD risk in the Korean population. We examined the incidence of CVD among 6,730 individuals who were initially free of CVD during a 17-year follow-up period. This study highlights the importance of integrating an advanced genetic risk assessment (multi-PRS) with traditional clinical tools (PCE) for more effective stratification of individual CVD risk.

## 2 Materials and methods

### 2.1 Study participants

This study was approved by the Institutional Review Board of the Korea Disease Control and Prevention Agency, Republic of Korea. In the Korean Genome and Epidemiology Study (KoGES), 10,030 participants were recruited from the Ansan and Ansung provinces (Kim and Han, 2017). Detailed descriptions of the KoGES have been provided in previous studies (Kim and Han, 2017; Moon et al., 2019). The participants (aged 40–69 years) provided written informed consent and were examined using epidemiological surveys, physical examinations, and laboratory tests. Of the 10,030 participants, 7,612 underwent genotyping and had at least one follow-up between 2001 and 2002 (baseline) and 2017–2018 (last follow-up). Individuals with a history of CVD or missing clinical risk factor data were excluded from further analyses of CVD incidence. Prevalent CVD cases were identified based on self-reports of IHD, stroke, heart failure, and myocardial infarction or taking medications related with CVD at the time of recruitment. Participants with past history of CVD were not used as prevalent cases to reduce possible recall bias in the subsequent analysis. Incident CVD cases were defined as individuals with CVD events during the follow-up period among the 6,730 participants initially free from CVD.

### 2.2 Genotyping and quality control

From the Ansan and Ansung cohorts (10,030 participants), 7,612 samples were genotyped using the Korea Biobank Array, a fully customized single-nucleotide polymorphism (SNP) microarray optimized for Korean genome research (Moon et al., 2019). The genotyping process and quality control measures have been described previously (Kim et al., 2022). Quality control included the exclusion of samples based on gender discrepancies, low call rates (<97%), excessive heterozygosity, second-degree related samples, and outliers in the results of the principal component analysis. Variants were excluded because of low call rates (<95%), Hardy–Weinberg equilibrium failure (*p* < 10^–6^), and low minor allele frequencies (MAFs) (<1%). After these exclusions, approximately 550 K SNPs remained for phasing and imputation.

### 2.3 Genotype imputation

Quality-controlled data were subjected to pre-phasing-based imputation. Eagle v2.3 (Loh et al., 2016) was used for phasing, followed by imputation using Impute v4 (Bycroft et al., 2018) with a merged reference panel of 2,504 samples from the 1,000 Genomes Phase 3 (Auton et al., 2015) and 397 samples from the Korean Reference Genome (Moon et al., 2019). The GEN-formatted file, an output from Impute v4, was converted to the VCF format with the imputed dosages using GEN2VCF (Shin et al., 2020). For further analysis, 8.3 M high-quality imputed common variants were retained, excluding those with imputation quality <0.8 and MAF <1%.

### 2.4 Calculation of the PRSs

PRSs for CVD were calculated using PRS-CS (Ge et al., 2019) with summary statistics from GWASs regarding myocardial infarction, ischemic stroke, and coronary artery disease conducted by Biobank, Japan (Sakaue et al., 2021). Due to the lack of publicly available, comprehensive summary statistics for hypertension PRS, we employed a ten-fold leave-one-group-out meta-analysis combined with PRS-CS analysis (Kim et al., 2022). The process involved the following detailed steps: 1) Randomly categorizing 125,850 Korean individuals into ten groups, 2) Conducting a GWAS for hypertension in each group, 3) Obtaining effect sizes and *p*-values from the meta-analyzed association results of nine groups, 4) Using PRS-CS to derive adjusted weights based on the meta-analysis results, 5) Calculating the PRS for the remaining one group using the weights obtained from the nine groups, and 6) Iterating these steps until PRS for all groups were obtained. Approximately 970 K HapMap phase 3 variants were used for the PRS calculations. The calculated PRSs were normalized to a normal distribution. Each PRS was weighted based on a multiple logistic regression model, with prevalent CVD cases (n = 59) as the outcome. The weighted sum of the PRSs (wPRSsum) was calculated for each individual. The participants were categorized into three genetic risk groups based on the wPRSsum: low (bottom 20%), intermediate (20%–80%), and high (80%–100%).

### 2.5 Calculation of the PCE score

The PCE was recalibrated for the Ansan and Ansung cohorts (Bae et al., 2020). This recalibration involved adjusting the equation to reflect the updated mean sum and baseline survival rate, accounting for the different sample sizes and extended follow-up periods in our study. The specific formulas used are as follows:

$$\begin{array}{ll} \text{PCEmen} & =4.950\times \mathrm{Ln}\left(\text{AGE}\right)+0.943\times \mathrm{Ln}\left(\text{TC}\right)\;–\;0.693\times \mathrm{Ln}\left(\text{HDL}\right)+1.101\times \mathrm{Ln}\left(\text{TRSBP}\right)\\ & +1.002\times \text{Ln(UNSBP)}+5.485\times \text{CUSMOK }–\;1.287\times \mathrm{Ln}\left(\text{AGE}\right)\times \text{CUSMOK}+0.558\times \text{DM} \end{array}$$ (1)

$$\begin{array}{ll} \mathrm{PCEwomen} & =36.699\times \mathrm{Ln}(\mathrm{AGE})+0.625\times \mathrm{Ln}(\mathrm{TC})–0.449\times \mathrm{Ln}(\mathrm{HDL})+29.947\times \mathrm{Ln}(\mathrm{TRSBP})–7.010\times \mathrm{Ln}(\text{AGE})\\ & \times \text{Ln}(\text{TRSBP})+29.255\times \text{Ln(UNSBP)}–6.847\times \text{Ln}(\text{AGE})\times \text{Ln}(\text{UNSBP})+0.497\times \text{CUSMOK}+\text{0.962}\times \text{DM} \end{array}$$ (2)

Abbreviations, as detailed in Supplementary Table S3 of Bae et al. (2020), are defined as follows: AGE: age, TC: total cholesterol, HDL: high-density lipoprotein, TRSBP: treated systolic blood pressure, UNSBP: untreated systolic blood pressure, CUSMOK: current smoking status, DM: status of diabetes mellitus.

The participants were then categorized into two risk groups based on their PCE scores: low (<7.5%) and high (≥7.5%).

### 2.6 Statistical analysis

A logistic regression model was used to assess the association between the PRSs or multi-PRS and prevalent CVD cases. The association between wPRSsum and/or PCE and incident CVD events was tested using a Cox proportional hazards model using the R package “survival,” adjusting for age and sex (Terry and Therneau, 2000). Kaplan–Meier curves were constructed and analyzed for incident CVD according to the risk groups based on PRS, PCE, and their combinations using the R package “survival.” The concordance index (C-index) was used to measure the accuracy of the predictive models based on the wPRSsum and/or PCE (Harrell et al., 1996).

## 3 Results

Among the 10,030 participants in the Ansan and Ansung cohorts, 7,612 were selected based on quality-controlled genotype data, non-missing clinical risk factors for PCE, and at least one biannual follow-up between 2001 and 2018. The characteristics of the participants are summarized in Table 1. The average and median follow-up durations were 13.31 and 16 years, respectively, and females constituted 51.6% of the study population. There were 59 prevalent and 759 incident cases of CVD.

**TABLE 1:** Demographic characteristics of the study participants.

	All	Men	Women
Number of participants, n (%)	7,612	3,675 (48.28)	3,937 (51.72)
Age (years)	52.06 ± 8.85	51.59 ± 8.72	52.49 ± 8.95
Female, n (%)	3,937 (51.72)	-	-
Follow-up, mean years (median)	13.31 (16.0)	13.09 (16.0)	13.51 (16.0)
Current smoker, n (%)	1,926 (25.30)	1,792 (48.76)	134 (3.40)
CVD prevalence, n (%)^a^	59 (0.78)	36 (0.98)	23 (0.58)
CVD incidence, n (%)^a^	759 (9.97)	370 (10.07)	389 (9.88)
SBP (mmHg)	117.41 ± 18.16	117.37 ± 16.73	117.44 ± 19.40
BP treatment, n (%)^b^	916 (12.03)	371 (10.10)	545 (13.84)
Diabetes mellitus, n (%)^c^	677 (8.89)	379 (10.31)	298 (7.57)
TC (mg/dL)	192.09 ± 35.93	192.72 ± 36.29	191.50 ± 35.58
HDL-C (mg/dL)	44.75 ± 10.08	43.68 ± 9.99	45.74 ± 10.05

^a^ Individuals with CVD, events during the follow-up period.

^b^ On hypertension-related medication.

^c^ Fasting plasma glucose ≥126 mg/dL or on type 2 diabetes mellitus treatment.

CVD, cardiovascular disease; SBP, systolic blood pressure; BP, blood pressure; TC, total cholesterol; HDL-C, high density lipoprotein cholesterol.

We employed the wPRSsum method, which combined the PRSs of myocardial infarction, ischemic stroke, coronary artery disease, and hypertension. The relationships between various PRSs and the 59 most prevalent CVD cases are presented in Table 2. Although, the hypertension PRS exhibited the strongest association with CVD, none of the single PRSs met the statistical significance threshold (*p* < 0.05). However, the wPRSsum, which incorporated all PRSs, demonstrated superior performance (OR = 1.39, *p* = 9.04 × 10^−3^) compared to the other combinations (OR < 1.33) and single PRSs (OR < 1.25), aligning with the findings of Lu et al. (Lu et al., 2021). Lu et al. developed a meta-PRS (multi-PRS) that included PRSs for various conditions such as lipids, blood pressures, type 2 diabetes, cardio arterial disease, and stroke, aimed at predicting the risk of future stroke. This multi-PRS approach demonstrated a significant increase in hazard ratio for stroke prediction, with CAD-PRS showing an HR < 1.2, compared to the multi-PRS’s HR of approximately 1.3, highlighting the incremental benefit of combining multiple PRSs. Mirroring this, our study found that the multi-PRS (wPRSsum in our case) presented an increased odds ratio (OR = 1.39) compared to single PRSs (OR < 1.25).

**TABLE 2:** Association between the PRSs and prevalent CVD.

PRS	Odds ratio	*p*-value
HTN	1.25	7.92 × 10^−2^
MI	1.18	1.52 × 10^−1^
IS	1.22	1.29 × 10^−1^
CAD	1.14	2.84 × 10^−1^
HTN + MI	1.27	4.31 × 10^−2^
HTN + IS	1.33	3.00 × 10^−2^
HTN + CAD	1.25	6.39 × 10^−2^
MI + IS	1.29	3.98 × 10^−2^
MI + CAD	1.23	9.75 × 10^−2^
IS + CAD	1.26	6.88 × 10^−2^
HTN + MI + IS	1.34	1.66 × 10^−2^
HTN + MI + CAD	1.32	2.40 × 10^−2^
HTN + IS + CAD	1.33	2.46 × 10^−2^
MI + IS + CAD	1.33	2.49 × 10^−2^
**HTN + MI + IS + CAD**	**1.39**	**9.04 × 10** ^**−3**^

PRS, polygenic risk score; CVD, cardiovascular disease; HTN, hypertension; MI, myocardial infarction; IS, ischemic stroke; CAD, coronary artery disease. The odds ratios were obtained using a logistic regression model for CVD-prevalent cases after adjusting for age and sex. The bold text in table indicates that the corresponding PRS model was used in this study.

**FIGURE 1:**
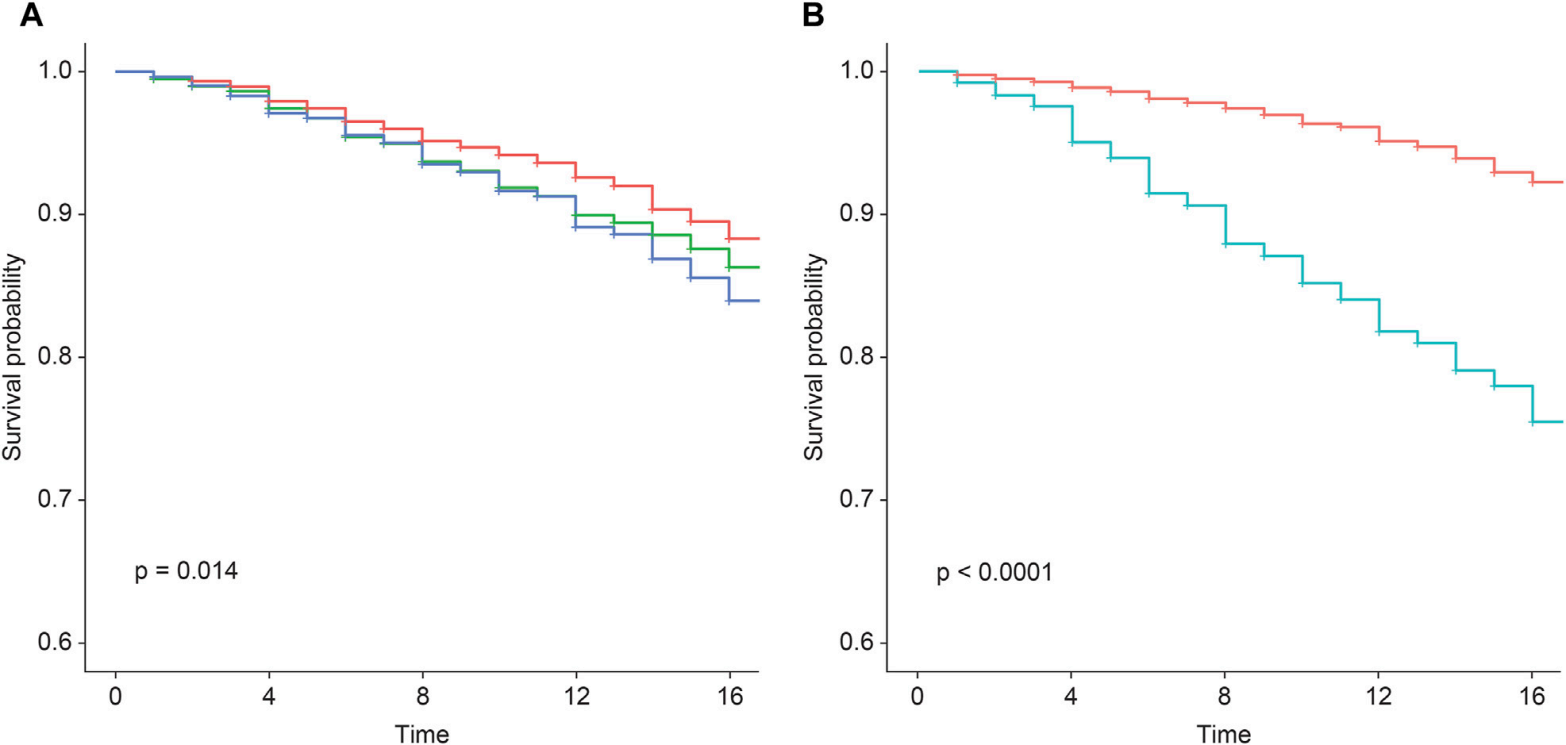
Survival rate regarding incident CVD according to the wPRSsum and PCE risk groups. Survival rate regarding incident CVD, stratified by **(A)** wPRSsum (bottom, 20% colored in red; intermediate, 20%–80% colored in green; top, 20% colored in blue) and **(B)** PCE (low, <7.5% colored in red; high, ≥7.5% colored in green). PRS (wPRSsum), polygenic risk score; CVD, cardiovascular disease; PCE, pooled cohort equations.

In assessing the 759 incident cases over 17 years, the wPRSsum showed a significant association with an HR of 1.15 (*p* = 7.49 × 10^−5^) (Table 3). The top 20% of the genetically high-risk group had an increased HR of 1.50 (*p* = 5.04 × 10^−4^) compared to the bottom 20%. The intermediate group (20%–80%) showed an HR of 1.28 (*p* = 1.26 × 10^−2^). Moreover, the Kaplan–Meier analysis indicated a distinct survival curve trend in the top 20% of the high-risk group (Figure 1A).

**TABLE 3:** Predictability of the wPRSsum for future CVD.

Group	N	Number of cases	Hazard ratio	SE	*p*-value
All samples	6,730	759	1.15	0.035	7.49 × 10^−5^
Low genetic risk (0%–20%) (baseline)	1,349	129	-	-	-
Intermediate genetic risk (20%–80%)	4,055	459	1.283	0.100	1.26 × 10^−2^
High genetic risk (80%–100%)	1,326	171	1.501	0.117	5.04 × 10^−4^

The association between PRS, and incident CVD, events was tested using a Cox proportional hazards model adjusted for age and sex.

PRS, polygenic risk score; CVD, cardiovascular disease; wPRSsum, weighted sum of the PRS; SE, standard error.

While the original PCE provides risk assessments for ischemic heart disease and stroke primarily for non-Hispanic African-American and non-Hispanic white populations aged 40–79 years, it does not directly cater to the specific risk of other racial groups (Goff et al., 2014). To address this limitation and incorporate racial considerations for the Korean population, we utilized the recalibrated PCE for the Korean population as reported by Bae et al., based on the Ansan and Ansung cohorts. This recalibration ensures that our cardiovascular risk assessments are appropriately tailored to our study population, reflecting the unique risk profiles of Koreans. When participants were divided into low (<7.5%) and high (≥7.5%) risk groups based on their calculated 10-year CVD risk, the high-risk group exhibited a significantly higher HR of 3.58 (*p* = 2.19 × 10–63) compared to the baseline low-risk group (Table 4). Moreover, the Kaplan–Meier analysis showed a marked decline in the survival curve of the high-risk group (Figure 1B).

**TABLE 4:** Predictability of the PCE for future CVD.

Group	N	Number of cases	Hazard ratio	SE	*p*-value
< 7.5% (baseline)	4,228	271			
≥ 7.5%	2,502	488	3.58	0.076	2.19 × 10^−63^

The association between the PCE, and incident CVD, events was tested using a Cox proportional hazards model adjusted for age and sex.

CVD, cardiovascular disease; PCE, pooled cohort equations.

**FIGURE 2:**
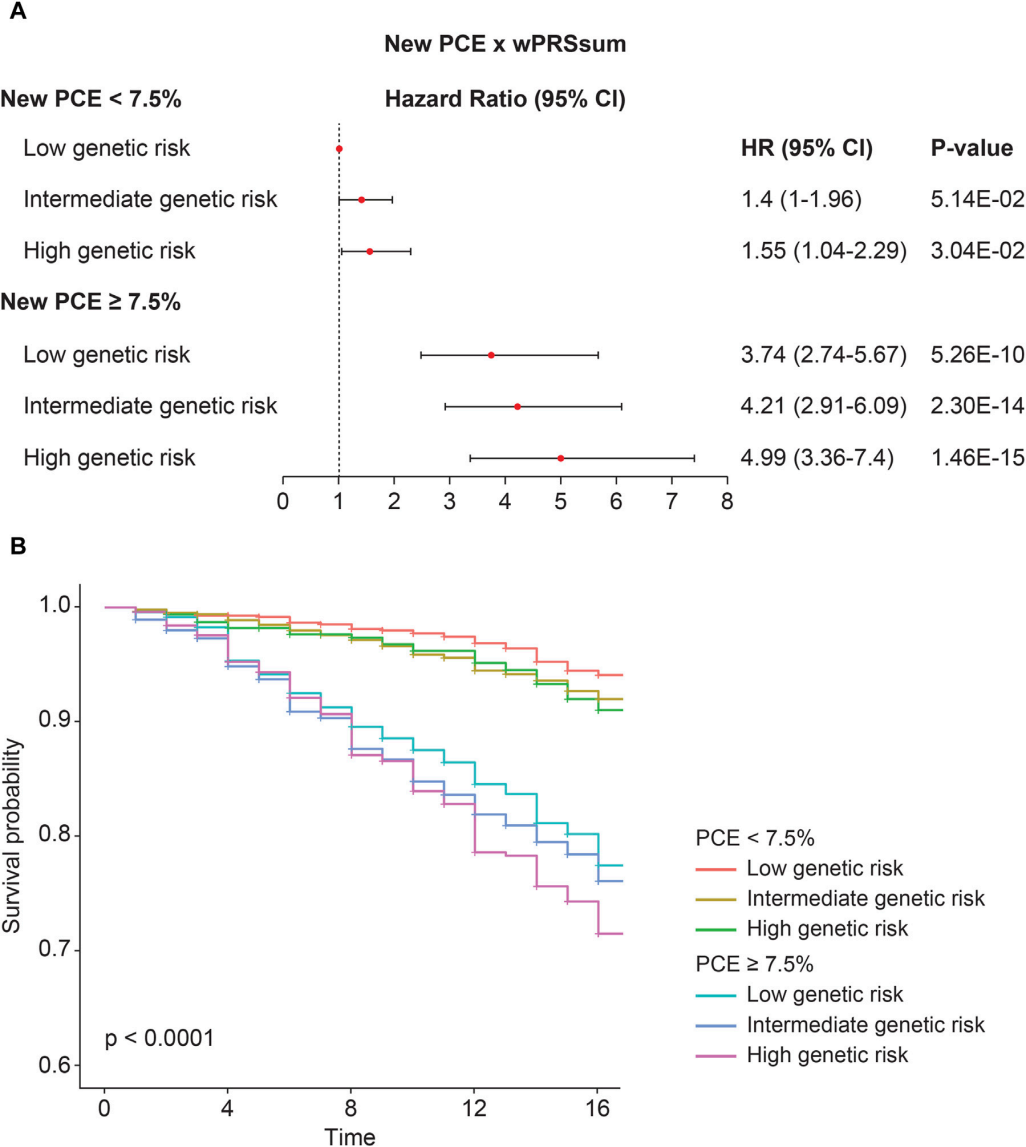
Predictability of the strata using wPRSsum and PCE. **(A)** Risk of future CVD according to the PCE and wPRSsum groups. Association of wPRSsum and PCE with incident CVD. **(B)** Survival rate of incident CVD, stratified by combinations of the wPRSsum (bottom, 20%; intermediate, 20%–80%; and top, 20%) and PCE (low, <7.5%; high, ≥7.5%). PRS (wPRSsum), polygenic risk score; CVD, cardiovascular disease; PCE, pooled cohort equations; HR, hazard ratio; CI, confidence interval.

Participants were stratified into groups based on both PRS and PCE risk levels. Compared with the baseline (low PRS and low PCE), there was an increase in the incident CVD risk with a higher PRS or PCE risk (Figure 2A; Table 5). A high PRS risk consistently showed an increased CVD risk, independent of the PCE group. PRS and PCE showed an increasing tendency toward CVD risk in a roughly additive manner. Moreover, the combination of high PRS and PCE risk yielded the highest incident CVD risk (HR = 4.99, *p* = 1.46 × 10^−15^). The Kaplan–Meier analysis showed a sharp decline in the survival curve of the high-risk group for both PRS and PCE (Figure 2B).

**TABLE 5:** Predictability of the strata by PCE and wPRSsum.

PCE (%)	wPRSsum	N	Number of cases	Hazard ratio	SE	*p*-value
**< 7.5**	Low (baseline)	868	42			
	Intermediate	2,544	169	1.399	0.173	5.14 × 10^−2^
	Top	816	60	1.546	0.201	3.04 × 10^−2^
≥ 7.5	Low	481	87	3.737	0.212	5.26 × 10^−10^
	Intermediate	1,511	290	4.211	0.188	2.30 × 10^−14^
	Top	510	111	4.986	0.201	1.46 × 10^−15^

The association between the combinations of PCE, and wPRSsum, and incident CVD, events was tested using a Cox proportional hazards model with a low risk in both PCE, and wPRSsum, as the baseline.

PRS, polygenic risk score; CVD, cardiovascular disease; PCE, pooled cohort equations; wPRSsum, weighted sum of the PRS.

Various models were constructed using age, sex, PRS, and PCE to predict the future CVD risk (Table 6). The five constructed models were as follows: model 1, age + sex; model 2, PRS; model 3, PRS + age + sex; model 4, PCE; and model 5, PRS + PCE. Model five did not include age or sex as variables because they were incorporated into the PCE equation. The models’ C-index scores were as follows: model 1 = 0.659, model 2 = 0.541, model 3 = 0.666, model 4 = 0.704, and model 5 = 0.703.

**TABLE 6:** Predictive performance of the models.

Model	All	Men	Women	Age <55	Age ≥55	Men		Women		Elliott et al. (2020)				
						Age <55	Age ≥55	Age <55	Age ≥55	All	Age <55	Age ≥55	Men	Women
Sample N	6,730	3,258	3,472	4,193	2,537	2,125	1,133	2,068	1,404	352,660	147,985	204,675	147,363	205,297
CVD incidence	759	370	389	311	448	163	207	148	241	13,753	2,854	10,899	8,595	5,158
AGE + SEX	0.659	0.650	0.666	0.600	0.531	0.572	0.500	0.625	0.545	0.70	0.68	0.65	0.65	0.68
wPRSsum	0.541	0.547	0.535	0.563	0.534	0.552	0.552	0.574	0.518	0.56	0.59	0.56	0.57	0.56
wPRSsum + AGE + SEX	0.666	0.658	0.672	0.618	0.542	0.589	0.553	0.647	0.549	0.71	0.70	0.66	0.67	0.69
PCE	0.704	0.691	0.717	0.676	0.607	0.636	0.599	0.714	0.613	0.72	0.73	0.67	0.68	0.71
PCE + wPRSsum	0.703	0.692	0.715	0.673	0.604	0.640	0.597	0.703	0.612	0.73	0.74	0.68	0.69	0.72

The C-index was assessed for the Cox proportional hazards models. CVD, prediction results of Elliot et al. were from Supplementary Table S1 of Elliot et al.

PCE, pooled cohort equations; wPRSsum, weighted sum of the polygenic risk scores.

When stratifying by gender and age (<55 and ≥55), model 5 (C-index = 0.692) slightly outperformed model 4 (C-index = 0.691) in males, while the opposite was true for females (0.717 and 0.715 for models 4 and 5, respectively) (Table 6). The incidence rates in the younger and older age groups were 7.4% and 17.7%, respectively (Table 6). However, in younger individuals, all models demonstrated higher C-index scores than in the older groups. Moreover, the addition of PRS to the PCE-only model was particularly beneficial in males aged <55 years (0.640 and 0.636 for models five and 4, respectively). Our investigation aligns with previous studies that have underscored the value of adding PRS information to clinical risk assessment tools for enhancing cardiovascular disease prediction. Notably, Elliott et al. reported a similar structure in their findings (Table 6). Specifically, our results reveal that adding PRS to the PCE model marginally improved the C-statistics for males (C-index = 0.691 for PCE alone and 0.692 for PCE + wPRSsum), with the most notable improvement observed in males under 55 years of age. This nuanced improvement, although modest, underscores the potential of PRS in enhancing risk stratification beyond the capabilities of PCE alone. It is important to note that our study observed improvements in specific subsets, particularly in younger males, rather than a uniform enhancement across all demographics, contrasting with Elliott et al., who reported more generalized improvements. This discrepancy highlights the complexity of cardiovascular risk prediction and the variable impact of genetic information across different population segments.

## 4 Discussion

This study evaluated the utility of multi-PRS (wPRSsum) and the PCE over a 17-year longitudinal study involving 7,612 Korean individuals. Our findings demonstrated that, although the linear model combining PRS and PCE did not significantly outperform the PCE-only model, the risk stratification approach indicated that the highest-risk group had an HR of 4.99. The PRS and PCE appeared to contribute additively to the CVD risk assessment.

Although PRS did not markedly improve the predictive performance of the CVD risk when combined with PCE, it did offer a marginal increase in prediction accuracy, particularly in males. This observation aligns with those of previous studies (Elliott et al., 2020; Ramírez et al., 2022; Patel et al., 2023; Yun et al., 2023). The limited improvement could be attributed to the robustness of existing clinical risk assessment tools (Elliott et al., 2020; Ramírez et al., 2022; Patel et al., 2023; Yun et al., 2023) and the partial inclusion of genetic components in the clinical factors used in the PCE construction. Nevertheless, previous studies have indicated improved discrimination and reclassification when the PRS is combined with clinical risk scores (Elliott et al., 2020; Ramírez et al., 2022; Patel et al., 2023; Yun et al., 2023). This study further supports this finding by revealing that stratification by PRS and PCE can identify individuals with a nearly five-fold increased risk of future CVD compared with the baseline risk group. Kaplan–Meier curves, coupled with 17-year incident data, revealed a significantly reduced survival rate in high-risk individuals according to both PRS and PCE. The complementary nature of the PRS and PCE suggests the potential for more personalized treatment strategies.

Our study validates previous findings and underscores the efficacy of using a stratification approach with PRS and clinical tools, such as PCE. One of the strengths of our study is its long follow-up period, which exceeded the average duration (13.31 years) used in previous studies (4.6–12.0 years) (Elliott et al., 2020; Lu et al., 2021; Ramírez et al., 2022; Patel et al., 2023; Yun et al., 2023). Additionally, this is the first application of the wPRSsum method in the Korean population, which demonstrated superior predictive performance compared with single PRS analyses, consistent with recent findings (Elliott et al., 2020; Lu et al., 2021; Ramírez et al., 2022; Patel et al., 2023; Yun et al., 2023). However, this study had some limitations. The recalibrated PCE tailored to the Ansan and Ansung cohorts might have led to overfitting, potentially overestimating the PCE’s performance in this study. The sample size, although substantial, was smaller than that of earlier studies (up to 380 K samples) (Elliott et al., 2020; Ramírez et al., 2022; Patel et al., 2023; Yun et al., 2023). Moreover, the CVD definition based on self-reported survey data may have introduced recall bias. Further research with larger cohorts and physician-diagnosed CVD or ICD-coded data is required for more accurate validation. Finally, the results may not be directly transferable to other ethnic groups because of population-specific optimizations in the risk assessment methods. Application in different populations requires suitable clinical tools and PRS models based on closely related ancestral GWAS data.

The prevalence of CVD and related risk factors varies significantly between males and females, as highlighted by the smoking rates of 50% for males and 3.4% for females in our cohort (Table 1). This pattern is somewhat mirrored in the African American cohort of the ARIC study, which informed the PCE construction, showing smoking rates of 37.3% for males and 24.0% for females (Goff et al., 2014). In this study, CVD incidence rates were 10.07% for males and 9.88% for females, respectively. Similarly, the ASCVD rates in the ARIC cohort were 11.1% for males and 7.2% for females. In constructing the PCE, variables were carefully selected to account for gender and racial difference (Goff et al., 2014). However, our analysis revealed that the predictive effectiveness of both PCE and PCE + PRS models was higher in women (C-index >0.7) than in men (C-index <0.7), aligning with findings reported by Elliot et al., where PCE and PCE + PRS showed greater benefits for women (Table 6). These observations suggest potential gender biases in the current models, indicating they may not be optimally configured for predicting CVD in males as compared to females. Developing more sophisticated models that consider these gender differences is crucial. However, the challenges of incorporating diverse populations for long-term risk assessment (beyond 10 years) remains a significant barrier to creating specialized models (Goff et al., 2014).

In conclusion, our study highlights the significant role of PRS and PCE in identifying individuals at a high risk of future CVD, working in a roughly additive manner. These insights contribute to our understanding of CVD etiology and may inform personalized prevention and treatment strategies for individuals with varying CVD risk levels.

## Data Availability

The original contributions presented in the study are included in the article, further inquiries can be directed to the corresponding author.
